# The Book World of Medicine and Science

**Published:** 1903-06-20

**Authors:** 


### THE BOOK WORLD OF MEDICINE AND SCIENCE.

INTERNATIONAL CLINICS. Edited by HENRY W. CATTELL, A.M., M.D., Phila. U.S.A. Vol. IV. Twelfth Series. (London: J. B. Lippincott Company. Pp. 317. With numerous Illustrations. 1903.)

THE design of a quarterly containing articles "by leading members of the medical profession throughout the world" is most excellent. We could desire that it had been possible for the Editor to have reached more closely to his original intention. The present issue, although bearing the proud title of "international" among its 24 contributors, has only five British, and perhaps none of these would even claim for himself the distinction of being "a leader." No less than 17 of the writers are connected with American institutions, and only two articles come from Europe. The various contributions vary much in value. In most instances they are manifestly intended for clinical lectures, and their value is ephemeral and the attraction mainly local. One feature is of interest in indicating how our English customs differ from the procedures and proprieties acceptable to our American cousins. Amidst the "scientific" material of the work are "biographical sketches of eminent living physicians," and a very realistic photograph shows "Dr. William W. Keen operating at the Jefferson Medical College" surrounded by white-robed assistants, crowds of note-book-in-hand students and—a charming nurse. All this is no doubt very attractive, but it is not our custom to enliven the tedium of our serious medical works by such thought-deviating illustrations, and hence a departure however desirable, from our traditions and ancient customs strikes us as peculiar. Another somewhat exceptional feature of the present issue is the devotion of one-third of the volume to a monograph on "The Blood in Health and in Disease," which, although excellent in many ways, is far from complete, and by no means up to date, and in several respects superficial and inconclusive. But while there is much to take exception to, there is, fortunately, much that is deserving of commendation. Dr. C. F. Gardiner publishes suggestive designs for a sanatory tent for use in the treatment of pulmonary tuberculosis. Mr. Stanmore Bishop deals with pain in abdominal diagnosis, and the illustrations accompanying the article should prove of considerable service. Dr. Purves Stewart has a chatty article on "The Diagnosis of Functional and Organic Hemiplegia." Mr. Moynihan details his experience regarding the surgical treatment of hæmatemesis from gastric ulcer, but adds nothing to the opinions which he has expressed in several papers recently published in this country. Ophthalmologists may be interested in Dr. Shoemaker's article on the clinical significance of binocular diplopia. The work is excellently printed, and the general get-up leaves nothing to be desired.

FIRST AID TO THE INJURED AND SICK. An Advanced Ambulance Handbook. By WARWICK and TUNSTALL. (Bristol: John Wright and Co. 1903. Third Edition. 240 pages. 240 Illustrations. Price 2s. 6d.)

IN this revised edition there are no important changes, and apart from minor alterations and corrections, there is little that is new. Some dozen illustrations have been added while a few have been improved. The book was originally written, we are told, to provide for the want of a concise and fairly well illustrated text-book on first aid. The authors point out that first aid should teach how to render temporary assistance by improvised means, and how a patient should be moved to a place of safety. We are further told that the scope of first aid is not to supersede medical or surgical treatment, but to render immediate temporary assistance in case of accident or sudden illness, before the doctor's arrival. The book contains many illustrations, which are very helpful in a work of this kind. But, while producing a well illustrated manual, we are disposed to think the authors have failed somewhat in their very commendable efforts to make it concise, and had simpler and fewer measures been described, the book would have been of greater service. We sought in vain for some reference to microbic infection of wounds. To those readers who have heard but little of micro-organisms, a few simple lines would have told all that is essential, at the same time enabling them and others to bring greater intelligence to bear in the application of first aid to wounds. The importance of the prevention of wound infection is not sufficiently insisted upon. In speaking of sterilising the hands, no mention is made of the nail-brush, which is after all one of the simplest and most efficient methods of providing against infection. It should be pointed out that, be the hands ever so clean in appearance, they should be scrubbed in soap and water before touching any wound. Though it appears a trivial matter, it is such a very important one, that in not mentioning the necessity of scrubbing the hands, in a work which has such wide uses, especially among the ambulance corps of the Kingdom, we think the authors have made a serious omission. Perhaps one of the best chapters is that dealing with the treatment of poisons, which is well arranged and thoroughly practical. As a supplement to lectures and demonstrations on first aid, the book will no doubt have valuable uses.

OPEN-AIR TREATMENT: ITS SIGNIFICANCE AND SOME PRACTICAL DIFFICULTIES. By ISABELLA MEARS, L.R.C.P.I. (Edinburgh and London: William Blackwood and Sons. Pp. 32. 1903.)

MRS. MEARS, in the introductory note to her readable brochure, expresses the opinion that the public are still so ignorant regarding the advantages of "open-air treatment" that "it seems desirable that some medical practitioners actively engaged in this special branch of practice should lay before them a few considerations," and this the writer proceeds to do in a manner which is attractive and reasonable, although somewhat superficial and lacking in originality and scientific suggestiveness. The pamphlet, however, is one which should at least in some measure attain its purpose, and add energy to the rapidly increasing force of public opinion which is calling for an extension of adequate measures for the efficient hygienic treatment of the tuberculous poor.

CITY OF LONDON DIRECTORY. 1903. (London: W. H. and L. Collingridge. Price 12s. 6d.)

WE have received a copy of this useful work of reference for 1903. It contains a Street Guide, Alphabetical Directory, Trades' Guide, Livery Companies' Guide, List of Liverymen Voters, a Biographical Directory, Corporation and Public Companies' Directories, and a coloured Map of the City of London showing the latest improvements and alterations in the City. There are Educational and Charitable sections of the book, giving most useful knowledge as to the numerous advantages which can be obtained within the City, many of which are little known. The Livery Companies' Guide is a very interesting and complete portion of the book, and an interesting feature is the Gallery of Portraits of City Notabilities, which is an innovation this year. Altogether, both for business and historical purposes, the "City of London Directory" is a most useful possession.

